# *CHST15* gene germline mutation is associated with the development of familial myeloproliferative neoplasms and higher transformation risk

**DOI:** 10.1038/s41419-022-05035-w

**Published:** 2022-07-07

**Authors:** Yi Chen, Yang Zhang, Zhihua Wang, Yewei Wang, Yujiao Luo, Nannan Sun, Shasha Zheng, Wenzhe Yan, Xiang Xiao, Sufang Liu, Ji Li, Hongling Peng, Yunxiao Xu, Guoyu Hu, Zhao Cheng, Guangsen Zhang

**Affiliations:** 1 Department of Hematology, Institute of Molecular Hematology, The Second XiangYa Hospital, Central South University, Changsha, Hunan China; 2 Department of Oncology, The Second XiangYa Hospital, Central South University, Changsha, Hunan China; 3 Department of Hematology, The First affiliated Hospital, Zhengzhou University, Zhengzhou, Henan China; 4 Department of Hematology, The Affiliated ZhuZhou Hospital of XiangYa Medical College, Central South University, Zhuzhou, Hunan China

**Keywords:** Cancer, Cell biology

## Abstract

Herein, we describe the clinical and hematological features of three genetically related families predisposed to myeloproliferative neoplasms (MPNs). Using whole-exome sequencing, we identified a *c.1367delG* mutation(*p.Arg456fs)* in *CHST15* (NM_001270764), a gene encoding a type II transmembraneglycoproteinthat acts as a sulfotransferase and participates in the biosynthesis of chondroitin sulfate E, in germline and somatic cells in familial MPN. *CHST15*defects caused an increased *JAK2V617F* allele burden and upregulated *p-Stat3* activity,leading to an increase in the proliferative and prodifferentiation potential of transgenic HEL cells. We demonstrated that mutant *CHST15* is able to coimmmunoprecipitate the *JAK2* protein,suggesting the presence of a *CHST15-JAK2-Stat3* signaling axis in familial MPN. Gene expression profiling showed that the *FREM1, IFI27* and *C4B_2* genes are overexpressed in familial MPN, suggesting the activation of an “inflammatory response-extracellular matrix-immune regulation” signaling network in the *CHST15* mutation background.We thus concluded that *CHST15* is a novel gene that predisposes to familial MPN and increases the probability of disease development or transformation.

## Introduction

Myeloproliferative neoplasms (MPNs) are a heterogeneous group of disorders including polycythemia vera (PV), essential thrombocythemia (ET), and primary myelofibrosis (PMF), that manifest, in most instances, in a sporadic form. However,familial clustering of MPN has been reported [1–3] and accounts for 5–10% of MPN cases [1, 3, 4]. Familial MPN is clinically indistinguishable fromsporadic MPN, and frequent somatic *JAK2* and *CALR*mutations exist [1, 3, 5]. Mutations in other germline genes, such as *TET-2* [6, 7], *TERT* [8, 9], *ATG2B/GSKIP* [10], *MECOM*, *HBS1L-MYB* [11], and *RBBP6* [12], may contribute to a small portion of familial MPN cases. However, the causative germline mutation in the majority of familial MPN cases is unknown. Independent acquisition of several oncogenic mutations in a single patient with MPN suggested that an unidentified germline predisposition locus might be present in some sporadic cases. The *JAK2* 46/1 haplotype with the *JAK2* mutation encoding *p.Val617Phe* was shown to increase the risk of developing MPN [13, 14]. Overall, the 46/1 haplotype has been estimated to account for 50% of the population attributable risk of developing an MPN in European populations, but it cannot explain and revealthegenetic predisposition factor for familial MPN inpopulations from different ethnic groups and geographical origins.

In the present study,we identified the *c.1367delG* (*pArg456fs*) mutation in the carbohydrate sulfotransferase 15(*CHST15*) gene in three Chinese families with MPN viawhole-exome sequencing. This mutation is responsible for earlier-onset PMF and the tendency toward progressive acceleration or transformation. We demonstrated that this*CHST15* mutation is associated with an increased *JAK2V617F* allele burden and upregulated*p-Stat3* activity. We also confirmed that this *CHST15* mutation endows MPN cells with increased proliferative activity and prodifferentiation potential, suggesting that this *CHST15* mutation is a candidate for predisposition to familial MPN.

## Materials and methods

### Patients and samples

Patients were diagnosed with PMF and ET according to the World Health Organization (WHO) criteria [15]. Familial cases were defined when two or more individuals in the same pedigree were affected with MPNs.Blood samples from familial patients (*n* = 6), family members (*n* = 9), patients with sporadic MPN (*n* = 110) and healthy subjects (*n* = 160) were collected after informed written consent was obtainedin accordance with the Declaration of Helsinki. This study was approved by the institutional ethics committee of The Second Xiang-Ya Hospital, Central South University. To detect germline mutations in the *CHST15* gene,oralmucosacells were retrieved frompatients with familial MPN (*n* = 6), patients with sporadic MPN (*n* = 30) and normal controls (*n* = 30) and were prepared with a Swab Pack (Isohelix, UK) as previouslydescribed [16]. *JAK2(V617F*) andMPN-specificsomatic mutations (*JAK2-V617F*, *MPL-W515L*,*CALR-type 1* and *JAK2* exon 12 mutation) were screenedin granulocyte DNA as previously described [17].

#### Whole-exome sequencingof germline and somatic cells

We performed whole-exome sequencing (WES) on germline and somatic cells fromthe threefamily members with MPN (*n* = 6).Genomic DNA was extracted from freshly peripheral blood mononuclear cells (PBMNCs) andoralmucosacells and was fragmentedand hybridized to capture arrays for enrichment. Exome capture sequencing was performed on the HiSeq 4000 platform by BGI-Tech with the paired-end 150 bp read option according to the manufacturer’s instructions (Wuhan, China). BWA software was used to align paired-end reads to the reference human genome (hg19, downloaded from http://genome.ucsc.edu/) with default parameters. After alignment, only uniquely mapped reads or confidently mapped paired-endreads were retained. Alignment calibration was performedby GATK, and Picard was used to mark duplicate reads from PCR. VarScan and SAM tools were employed to detect somatic variations or insertion/deletions (INDELs), and the data were processed by further filtering to eliminate false positives.

### All mutations were annotated with ANNOVAR

#### Gene expression profiling and data analysis

Blood samples were collected from six patients with familial MPN (PMF, 4; ET, 2), their family members (*n* = 2 for ET), and four patients with sporadic MPN (PMF, 2; ET, 2), and total RNA wasextracted. Twelve sampleswere analyzedon the BGISEQ-500 platform,and a total of 17,610 genes were assayed. Microarrayhybridization was performed according to the manufacturer’s protocols. Differentially expressed genes between twosamples were identified through fold-change filtering (≥2.0). Pathway analysis was performed with gene mapping to KEGG pathways (*P* < 0.05). Pathway and GOanalyses were applied to determine the functions of differentially expressed genes in these biological pathways or GO terms. Finally, hierarchical clustering was used to distinguish gene expression among samples.

#### Analysis of the *JAK2V617F* mutational burden and phosphorylation of*Stat3/5* as well as *p38*

DNA was extracted from fresh frozen PBMNCs in samples from 5 patients with familial MPN and 50 patients with sporadic MPN using a TIANamp Genomic DNA Kit (Tiangen, China). The *JAK2V617F* mutant allele burden was measured by quantitative real-time PCR(qRT-PCR) as previously described [18]. Amplification was performed in a Rotor Gene real-time PCR 6000 system (Corbett Research, Wasserburg, Germany). Genomic DNA (20 ng) was used as the template. Primer sequences flanking the mutated region were employed with TaqMan probes (the sequences are available insupplemental materials (1)). The amount of the *JAK2V617F* allele was calculated by comparison with serial dilutions of mutant DNA obtained from a PMF patient with amutant allele percentage of 100% and wild-type DNA obtained from healthy individual. The mean of triplicate ΔCT determinations (CT *JAK2V617F*–CT *JAK2WT*) was used to calculate the mutant allele percentage. Usingflow cytometry, assays of *p-Stat3,p-Stat5 and p-p38* protein abundancesinPBMNCs from patients with familial MPN (*n* = 5) and sporadic MPN (*n* = 15) were performed.

#### Immunoprecipitation

Immunoprecipitation was performed with wild-type *CHST15* and mutant *CHST15* in HEL cells and PBMNCs from patients with familial MPN (*n* = 6),sporadic MPN with *JAK2V617F* mutation (*n* = 3), MPN with *CALR* mutation (*n* = 3), and triple-negative MPN (*n* = 3). The cells were lysed in RIPA lysis buffer. Before immunoprecipitation, the samples were precleared by adding 20 μL of Protein A/G PLUS-agarose (Santa Cruz,, Texas) and 1 μg of control IgG. Lysates were incubated overnight at 4 °C with anti-*JAK2* or anti-Flag-*CHST15* antibodies, as well as with anti-*Calnexin*, anti-*Cyclin B1*, anti-*HCK* and *anti-IFI27* antibodies. Affinity beads were collected and washed with immunoprecipitation buffer, and bound proteins were eluted. Eluates were resolved by SDS-PAGE and immunoblotted with specific antibodies.

#### Confocal immunofluorescence microscopy

Flag (3 × Flag)-tagged wild-type *CHST15* and mutant *CHST15* were cloned into the CV548 vector (Genechem, Shanghai, China). HEL cells were plated and cultured in RPMI 1640 medium supplemented with 10% FCS. Cells were transfected using Liposomal Transfection Reagent (Hanbio, Shanghai, China) with 4.0 µg of 3 × Flag-*CHST15*WT or 3 × Flag-*CHST15* Mut plasmid as well as 2.0 µl of transfection reagent. Transfected cells were selected in medium containing 2 µg/ml puromycin (Thermo Fisher Scientific, USA). After the cells were washed, fixed (4% paraformaldehyde) and permeabilized (0.1% Triton X-100), a blocking step was performed at room temperature (4% bovine serum albumin in PBS). Then, the cells were washed with 1 × PBS and incubated with anti-FLAG (mouse mAb, Sigma–Aldrich) and anti-*JAK2* (rabbit mAb, Cell Signaling) antibodies overnight at 4 °C. After washing, the cells were stained with an Alexa Fluor 488-conjugated anti-mouse antibody and an Alexa Fluor 594-conjugated anti-rabbit antibody (Invitrogen) for 1 h and were then incubated in DAPI for 1 h at 37 °C. Finally, the cells were visualized and imaged using a confocal laserscanning microscope (Zeiss, Jena, Germany).

#### Other methods and materials

The details of the other methods used in this study, i.e. colony formation experiment, RNA extraction, cDNA synthesis, and qRT-PCR of CHST15 mRNA, lentiviral vector constructs and CHST15 gene transfection, differentiation markers analysis of CHST15 mutants, Immunoblotting assay, Wright-Giemsa staining [19]. Additional information concerning the sequences of primers for PCR amplification or Sanger sequencing are available in theOnline Supplementary Materials.

### Statistical analyses

Quantitative data are presented as the mean ± standard error ofmean (SEM). Comparisons between groupswere made with one-way ANOVA or a Mann–Whitney Utest. Statistical differences were considered significant at *P* < 0.05.

## Results

### Clinical characteristics

**Fig. 1:**
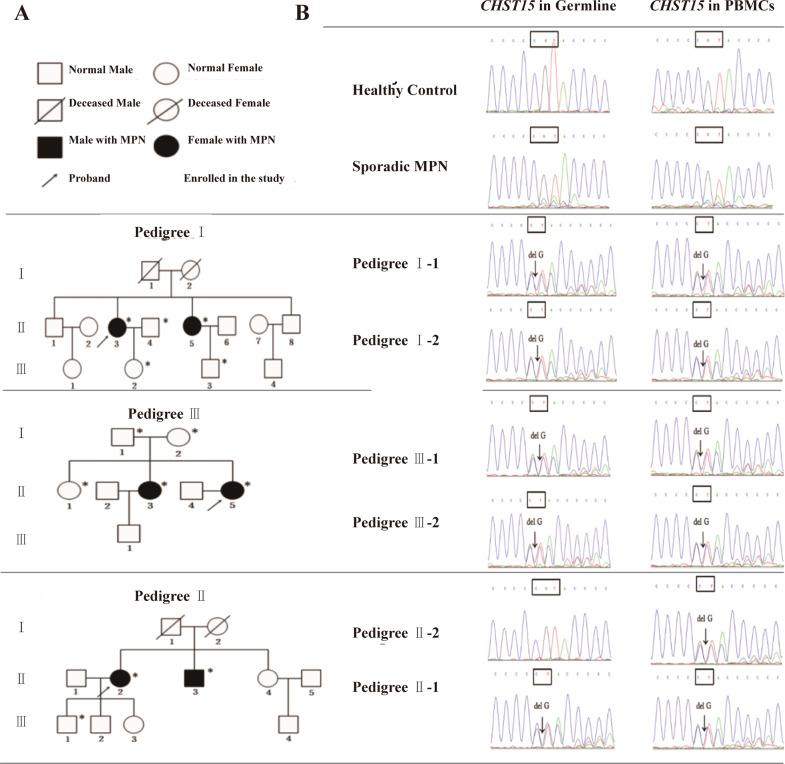
Pedigrees of the three families MPN and identification of somatic and germline mutation in *CHST15* gene. **A** Pedigrees of familial MPN. Filled symbols represent affected cases. Oblique line indicates the proband; PMF, primary myelofibrosis; ET, essential thrombocythemia. **B** Sanger sequencing of *CHST15* mutation both the somatic cells and the germline cells.

Our study cohort included 6 patients with MPN with germline *CHST15* mutations from 3 unique families.There were 5 females and 1 male; the age at disease onset ranged from 28 to 55 years, with a median age of 46.3 years. The course of the disease was 6 to 13 years, with a median survival periodof 9.67 years. The patients were followed - upcontinuously for 7–12 years.The patients experienced either malaise with left upperabdominal discomfort (4 PMF patients) or fatigue (2 ET patients). Additional testing revealed the presence of the *JAK2V617F* mutation in 5 cases and the *CALR* gene mutation in 1 case. Upon evaluation, 2 patients (Family 1) had moderate anemia (PMF), and 5 patients displayed an increased platelet count (range: 342–1100 × 10^9^/L).All PMF patients were found to have splenomegaly with the spleen tip 3–5 cm below the left umbilical margin. Bone marrow biopsy showed increased megakaryocytes and strong reticulin staining (MF-3). Concurrent cytogenetic analysis revealed normal karyotypes.Six years after diagnosis, doubleframeshift mutations at two loci in *ASXL1* (*c.1934dup, p.Gly646TrpfsTer12 and c.2428_2431del, p.Asp810MetfsTer7*) were found in one patient with PMF (family 2) during screeningfor MPN-related genes. One patient was found to have a mutation in *TET2 (p.Asn427ValfsTer4)* (family 1) and *TP53 (p.Lys164AsnfsTer6*) mutation and has gotten into MDS-EB-II, then, transformed into AML and did of severe pneumonia. No patient experiencedthromboembolic events, and 3 (PMF) had an accelerated phase. Leukemic transformation occurredin two patients (family 1:proband 1; duration:12 years; Family 1; proband’s young sister; duration 12 years), proband 1 harbouring *EZH2* (c.707delC (p.Thr236fs mutation experienced erythropoietic failure (normoblasts:2%) with severe anemia. Four months after treatment with ruxolitinib, the disease evolvedto myelodysplastic syndrome (MDS-EB-II, 13% myeloblasts) and transformed into acute myeloid leukemia; the patient finally died of infection and bone marrow failure on March 15, 2018. Notably, this case went through a dramaticevolution in that the original PMF transformed into PV and thenreturned to PMF until its evolution into MDS/AML. Another caseof PMF (family 1) has entered MDS-EB-II stage (17% myeloblast) at present. More interestingly, the two sisters suffered from the same MPN subtype, shared the same *CHST15* mutation and undergone a similar transforming courses. A patient with ET (family 3) was complicated bycarcinoma of the uterine cervixin May 2019 (Fig. 1A). The characteristics of the *CHST15* mutations in 6 patients with MPN are shown in Table 1.

**Table 1:** Characteristics of *CHST15* mutants in familial MPNs.

Family	Cases	Age of onset	Sex	diagnosis	Germline mutation	Somatic mutation	karyotype	MPN specific mutation	Additional mutation	Courses	Outcome
Family 1	1	54	F	PMF	*pArg456fs*	*pArg456fs*	Normal	*JAK2V617F*	*EZH2(c.707delC(p.Thr236fs)*	12 years	Transfomed AML, death
Family 1	2	52	F	PMF	*pArg456fs*	*pArg456fs*	Normal	*JAK2V617F*	*TET2(p.Asn427ValfsTer4);TP53(p.Lys164AsnfsTer6)*.	12 years	PMF-MDS-EB-II-Transformed AML, death
Family 2	3	40	M	PMF	*pArg456fs*	*pArg456fs*	Normal	*JAK2V617F*	*ASXL1(p.Gly646TrpfsTer12)*; *ASXL1* (*p.Asp810 MetfsTer7*.)	11 years	PMF-accelerated
Family 2	4	55	F	PMF	*pArg456fs*	Negative	Normal	*JAK2V617F*	No	9 years	PMF-accelerated
Family 3	5	28	F	ET	*pArg456fs*	*pArg456fs*	Normal	*JAK2V617F*	No	14 years	stable
Family 3	6	49	F	ET	*pArg456fs*	*pArg456fs*	Normal	*CALR L367fs*	NA	7 years	Complicated bycarcinoma of uterine

### Discovery of mutations through whole-exome sequencing

We captured and sequenced exomes from PBMNCs and oralmucosacellsfrom patients with familial MPN (*n* = 6). A recurrent germline and somatic mutation in *CHST15* (*c.1367delG, p.Arg456fs*) were found in all six patients and five patients, respectively. The prevalence of this mutation in the general population is 0/60 706 (in Exome Aggregation Consortium database). This mutation was not found in 160 healthy subjects or 110 patients with sporadic MPN. It is noteworthy that most patients (5/6) exhibited a canonical somatic mutation in *CHST15* (*c.1367delG, p.Arg456fs*), except in one patient, who wasthe proband’solder sister in family 2 (*germline mutation in CHST15 (c.1367delG, p.Arg456fs)* was positive, *CHST15*somatic mutation wasabsent). Germline and somatic *CHST15* mutations were confirmed by Sanger sequencing (Fig. 1B). In addition, family 1’s daughter and son, family 2’s son and daughter, and the proband’s parents in family 3 did not have both germline and somatic mutations. We also detected the germline *CHST15pArg456fs* mutation in 34 patients with sporadic MPN, and no mutations were found P) (Fig. 1B).

### Familial MPN gene expression signatures involved in extracellular matrix and immune responses

**Fig. 2:**
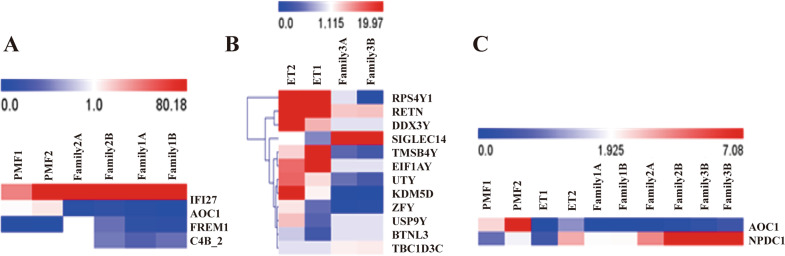
Familial PMF with *CHST15* mutation exhibits activation of immune/inflammation/extracellular matrix signals in PBMNCs. **A** Heatmap shows hierarchical clustering of expression profiles of 4 differentially expressed *CHST15* target genes in familial PMF (red:upregulation;blue:downregulation). Microarray assay of genes with >2.0-fold upregulation or downregulation. **B** Familial essential thrombocythemia (ET) genes expression profiles. Heatmap shows hierarchical clustering of profiles of 12 differentially expressed genes. **C** Genes expression profiles both in familial PMF and in familial ET.

Employing gene expression profiling assays, we identified 4 genes whose expression was significantly upregulated or downregulated in familial PMF. Among these genes, *ERAS1*-related extracellular matrix 1(*FREM1*) (*p* = 0.043), interferon alpha inducible protein 27(*IFI27*) (*p* = 0.032) and complement component 4B (*C4B_2*) (*p* < 0.0001) displayed obviousupregulation; amine oxidase copper domain-containing protein 1(*AOC1)* (*p* < 0.0001) was significantly downregulated (Fig. 2A). Relative to their expression in patients with sporadic ET, 12 genes were differentially expressed in 2 familial ET patients (Fig. 2B). These genes were mainly involved in *RET* signaling (*SIGLEC14*), *ERK* signaling, the actin cytoskeleton (*TMSB4Y*), cytokine receptor interaction signaling (*RETN*) and metabolism-related signaling (*KDM5D, UTY*). Gene expression profiles in both familial PMF and familial ET were integrated and compared mutually with those in sporadic PMF or ET. The results indicated that the expression of the neural proliferation differentiation and control protein 1 (*NPDC1*) gene was upregulated and the expression ofthe *AOC1* gene was downregulated in familial MPN (Fig. 2C).

### CHST15 mutation is complicated by upregulated JAK2V617F allele burden and enhanced p-STAT3 signaling

**Fig. 3:**
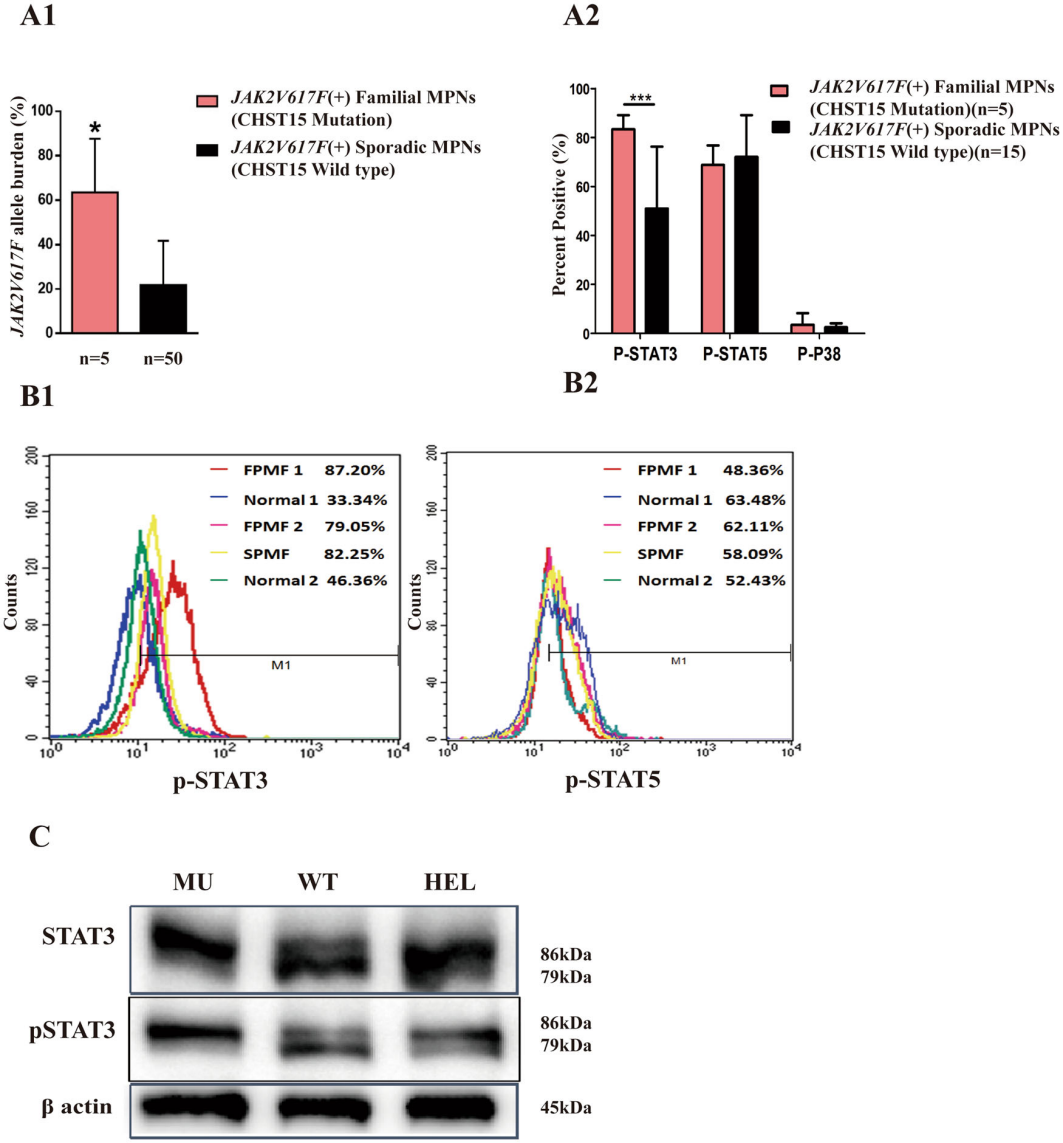
Effects of *CHST15* mutation on *JAK2V617F* allele burdens and proteins expression of *p*-*stat3*, *p*-*stat5* and *p-p38* on PBMNCs from familial MPNs (*n* = 5), sporadic MPNs (*n* = 50). **A1** Histograms show significantly increased *JAK2V617F* allele burdens in familial MPNs with *CHST15* mutation (*p* = 0.016). **A2** Histograms indicate upregulated *p*-*stat3* expression in familial MPNs corresponding to sporadic MPNs (*n* = 15) (*p* < 0.001); but no statistic difference is found for *p*-*state5* and *p-p38* between familial MPNs and sporadic MPNs. **B1**/**B2**: A representative FCM figures for *p-stat3* and *p-stat5* assays. Data are presented as mean ± s.e.m. ***p < 0.001; *p < 0.05, Student’s *t* test. **C** Western blot results show that cells harbouring *CHET15* mutation present upregulated expression of *p-stat3*.

We determined the effects of *CHST15* mutation on JAK2 mRNA and *stat* signaling. The *JAK2V617F* mutant allele burden (${{{\bar{\mathrm x}}}}$ = 63.48% ± 24.15%)in 5 familial MPN patients (*JAK2V617F* positive) was significantly higher than that in patients with sporadic MPN (*n* = 50; ${{{\bar{\mathrm x}}}}$ = 21.84% ± 19.85%) (*p* = 0.016) (Fig. 3A). Flow cytometric assays showed that the*p*-*Stat3* protein level (MFI: 83.36%) was obviously higher in familial MPN thanin sporadic MPN (*n* = 15) (MFI: 50.98%) (*p* < 0.001). There was no significant difference in *p-Stat5* and *p-p38*levels between familial MPN and sporadic MPN (Fig. 3A, B). The western blot results demonstrated that cells harboring the*CHET15* mutant had increased levels of *p-Stat3* (Figs. 3C, S1).

### Familial PMF patients and HEL cells harboring the *CHST15* mutant display downregulated *CHST15* protein and mRNA expression

**Fig. 4:**
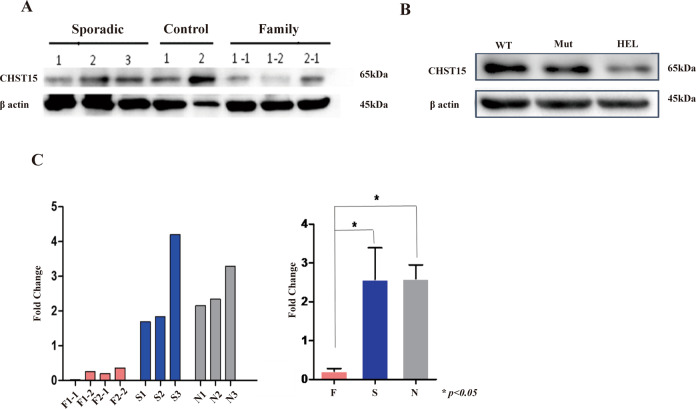
*CHST15* protein and mRNA expression in familial MPN patients and HEL cells with *CHST15* mutant (Western blot results). **A** from left to right, *CHST15* blotting bands represent sporadic MPN patients; healthy controls and familial MPN, respectively. **B** *CHST15* protein expression on HEL cells with wild-type *CHST15* (WT) and mutant *CHST15* gene(Mut) and non - transfected HEL cells (HEL). **C** Quantitative RT-PCR analysis of *CHST15* mRNA. Left panel: from left to right, F1-1, F1-2, F2-1, F2-2 indicate familial PMF; S1, S2, S3 indicate sporadic PMF; N1, N2, N3 indicate normal individuals. Results are means of three independent experiments. Right panel: when the mRNA expression levels were compared by pairwise comparison, the results showed that there was significantly depressed expression on *CHST15* mRNA in familial MPN (*p* < 0.05).

To test whether the*CHST15* mutation influences the protein and mRNA expression of *CHST15*, western blotting and quantitative real-time PCR(qRT-PCR) were performed. The results showed that compared with sporadic PMF patients, patients with familial PMFexhibited significantly decreased *CHST15* expression (Figs. 4A, S2), consistent with HEL cells harboring mutant *CHST15* (Figs. 4B, S3). The qRT-PCR results indicated that *CHST15* mRNA expression was obviously decreased in patients with familial MPN compared with patients with sporadic MPN and normal subjects and there were significant difference between familial MPN and sporadic MPN or normal controls (*p* < 0.05) (Fig. 4C).

### The *CHST15* variant confers a proliferative advantage and triggers the differentiation of HEL cells

**Fig. 5:**
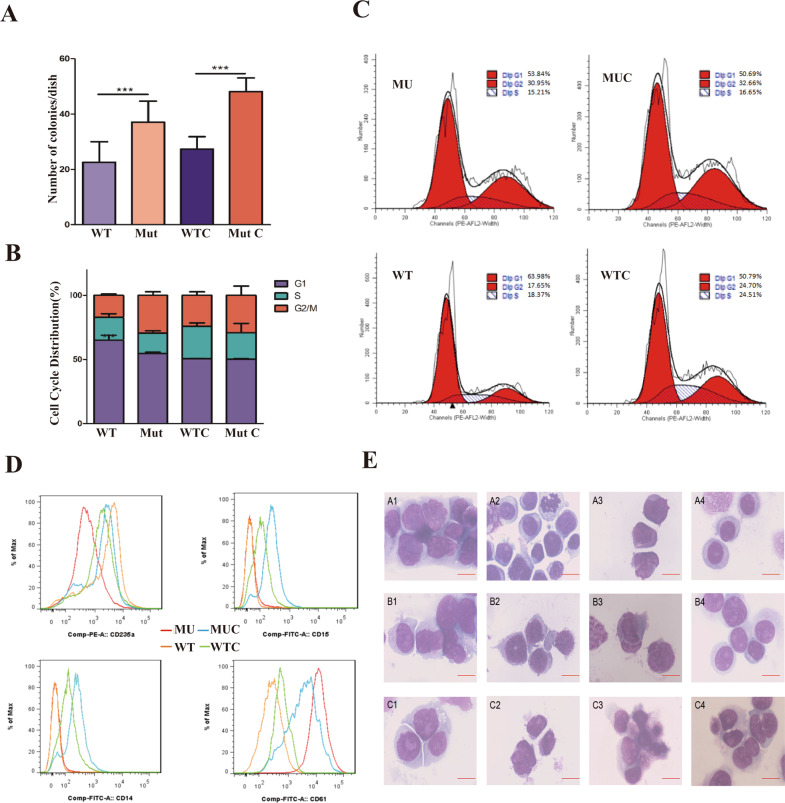
Functional analysis of *CHST15* mutant. **A** *CHST15* mutant promotes HEL cells proliferation with cytokine-independent fashion (colony-forming tests): HEL cells harbouring *CHST 15* mutant (1 × 10^5^) or wild-type *CHST15* (1 × 10^5^) were plated under semisolid condition and colonies were counted 14 d after cultured. Shown are average colonies ± s.e.m; The experiment performed in triplicate. ***p < 0.001. **B** Analysis of cell cycle distribution: HEL cells with *CHST15* mutant (MU) or *CHST15* mutation-negative (WT) in the presence (WTC or MUC) or absence of cytokine were cultured in 1640 medium; cells were harvested at day 7 and were analyzed by flow cytometry. Compared with WT, HEL cells with *CHST15* mutant showed an increased proliferative index (S phase plus G2/M phase%) (***p < 0.001), and independent of growth factors. **C** A representative image for cells cycle analysis. **D** Detection of differentiation markers by flow cytometry. demonstrated upregulation of cell surface markers CD14, CD15 and CD61 in HEL cells with *CHST15* mutant in the presence of cytokines; whereas there was no effecton CD235a expression. Data are shownas the mean ± s.e.m of three independent experiments. **E** Morphological changes associated with differentiation of HEL cells were evidenced by Wright-Giemsa staining (100×). Upper panel: A2, A4 showed characteristics of myeloid differentiated in *CHST15* mutant with shrunken cell nucleus and decreased the nuclear/cytoplastic ratio (N:C ratio) (A1-WT; A2-MU; A3-WT plus cytokine; A4-MU plus cytokine); Middle panel: B1, B4 represented monocytic differentiation with alleviated nuclear- folded and regular cellular outline in *CHST15* mutant cells. (B1-WT; B2-MU; B3-WT plus cytokine; B4-MU plus cytokine); Lower panel: C1, C4 represents megakaryocytic differentiation (C1-WT; C2-MU; C3-WT plus cytokine; C4-MU plus cytokine).

We then sought to determine whether the *CHST15* variant was associated with the disease phenotype. HEL cells, a human erythroleukemia cell line harboring the *JAK2V617F* mutation, were used as a cellular model, and cultured in the presence or absence of GM-CSF, G-CSF, EPO, SCF, and IL-3.The clone forming experiments showed that both*CHST15-WT* and *CHST15-Mut* cells displayed cytokine-dependent growth,In the absence of growth factors, *CHST15-Mut*cells had stronger proliferative activity (*p* < 0.001), suggesting the growth manner was cytokine-independent (Fig. 5A). Cell cycle analysis showed that in the absence of cytokines, *CHST15-Mut* cells displayed an increase in proliferative index (S- and G2/M-phase populations) (*p* < 0.05) compared to those populations of *CHST15-WT* cells (Fig. 5B, C), implying that the *CHST15* mutation overcomes G1 phase cell cycle block, resulting in enhanced growth potential.

We then investigated whether *CHST15* mutation contributes to HEL cell differentiation.We measured the expression of myeloid (CD15, CD14), erythroid (CD235a) and megakaryocytic lineage (CD61) markers by flow cytometry. Notably, the results showed that *CHST15* mutation enhanced the expression of CD15, CD14 and CD61, and this enhancementwas more evident in the presence of cytokines (Fig. 5D), suggesting that the *CHST15* mutant endows HEL cells with increased differential potential toward the myeloid or megakaryocytic lineage. Wright-Giemsa staining confirmed that *CHST15* mutation strikingly reduced the nuclear:cytoplasmic ratio (N:C ratio) compared to that of control HEL cells (Fig. 5E).

### *JAK2/CHST15* complex formation

**Fig. 6:**
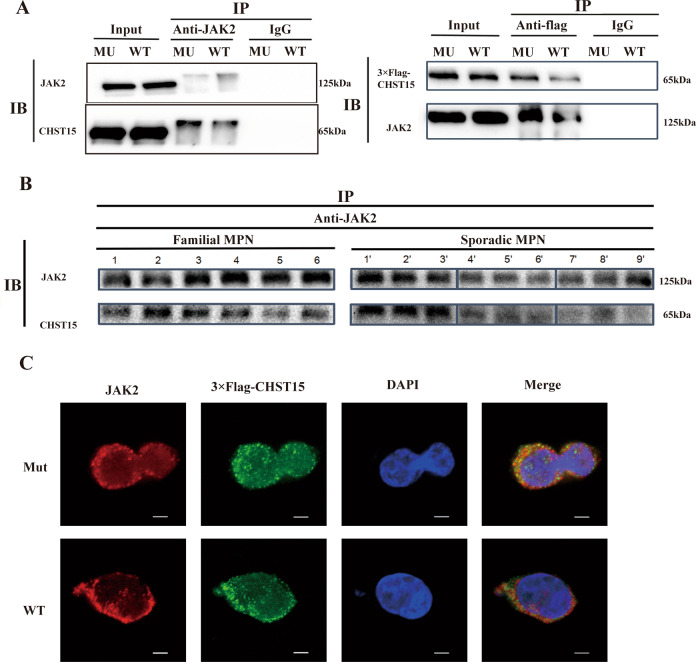
JAK2/CHST15 complex formation. **A** Coimmunoprecipitation between *JAK2* and *CHST15* on HELcells harbouring *CHET15* mutation (*CHST15*^R456fs^) or wild-type *CHST15*. left panel:*anti-JAK2* antibody; right panel:anti- Flag-*CHST15* antibody. Input, non-immunoprecipitated cell lysates; IgG, control IP with isotype antibody. **B** Coimmunoprecipitation between *JAK2* and *CHST15* on primary PBMNCs of patients with familial MPN (left panel: 1, 2, Familial 1. PMF: 3, 4. Familial 3. ET; 5,6. Familial 2, PMF.) or sporadic MPN (right panel: 1’, 2’, 3’: sporadic MPN with *JAK2V617F*; 4’, 5’, 6’: MPN with *CALR* mutation; 7’,8’,9’: MPN without mutation- triple negative). **C** Immunofluorescent colocalization of FLAG-tagged-*CHST15* and *JAK2*. 4′,6-diamidino-2-phenylindole (DAPI) wasused for nuclear staining. The results show that both *JAK2* and *CHST15* are mainly found on cytomembrane and cytoplasm, and form co-precipitatation with granular shape between *CHET15* and *JAK2*, suggesting exists in physical interaction both *JAK2* and *CHST15* (wild type) especially *CHST15*^*R456fs*^ mutant.

We performed coimmunoprecipitation experiment on bothprimary PBMNCs from patients with familial MPN and HEL cells. The results showed that *JAK2* coimmunoprecipitated with *CHST15* in HEL cells (Fig. 6A, left); conversely, *CHST15* directly interacted with *JAK2* (Fig. 6A, right, Fig. S4). However, *CHST15* could not be coimmunoprecipitated with *calnexin*, *cyclin B1*, *Hck*or*IFI27* (data not shown). In PBMNCs from patients with MPN, *JAK2* interacted with *CHST15*. However, there were some differences in the strength of the interaction, The abundance of *CHST15* in familial MPN harboringthe *CHST15* mutation was lower than that in sporadic MPN. In addition, the abundance of *JAK2* in familial MPN washigher than that in sporadic MPN (Figs. 6B, S5), suggesting an attenuated inhibitory effect of the *CHST15* mutant on *JAK2*. The interaction between *CHST15* and *JAK2* was also confirmed by the acquisition of fluorescence images using a confocal laser scanning microscope, showing that *JAK2* was mainly distributed on the plasma membrane and in the cytosol and nucleus, while *CHST15* was mainly located onthe plasma membrane and in the cytosol. Compared with its location in wild-type cells, the *JAK2/CHST15* complex was mainly localized on the inner side of the cell membrane in cells expressing the *CHST15* mutant (Fig. 6C).

## Discussion

**Fig. 7:**
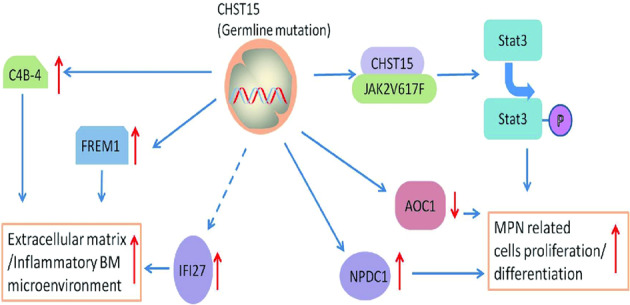
A diagram of gene regulatory network for describing the possible mechanism in familial MPN with germline CHST15 mutation resulting in the dysregulation of MPN cells proliferation, differentiation and BM microenvironment unbalance. Upward arrows: upregulated; Down arrow: downregulated.

We identified *CHST15* germline mutationscoexisting with somatic mutations that predispose to PMF and ET in three Chinese families. Our familial MPN cases had the same geographical origin, with an earlier MPN onset in comparison to sporadic cases (46.3 years versus 58.5 years [6]), an obvious female predominance and a tendency toward progressive transformation to an accelerated phase of PMF or acute myeloid leukemia. Consistent with theclinical features, our results showed that patients with the *CHST15* mutation exhibited an increased *JAK2V617F* allele burden and activated *p-stat3* signaling, suggesting that the *CHST15* mutation cooperateswith the classical *JAK2* V617F mutation to trigger the MPN phenotype. To explore the consequences of *CHST15* mutation, we also established transgenic cell models. The subsequent results indicated that HEL cells harboring the *CHST15* mutation displayed overt proliferative activity with a cytokine-independent pattern, implying that this mutation increases the sensitivity of myeloid or erythroid progenitors to hematopoietic growth factors. More importantly, we observed that the *CHST15* mutation might induce HEL cell differentiation toward myeloid, monocytic and megakaryocytic lineages, which mimicked the bone marrow morphologic characterization ofPMF or ET, showing an enhancing effect of *CHST15* mutation on MPN development (Fig. 7). Notably, when additional epigenetic events were detected during follow-up, 3 patients with PMF were found to have mutations in *IDH1*,

*EZH2, ASXL1*, *TET2* and,*TP53* mutation. Consistent with these changes, in the 3 patients with coexisting germline and somatic *CHST15* mutations, the disease converted to acute myeloid leukemia or accelerated PMF, illustrating that *CHST15* mutation can enhance the transformation potential of PMF.

Familial segregation of hematological malignancies supports the role of inherited factors in disease etiology [20, 21]. Direct evidence for a predisposition to MPNs is provided by the increased risk related to MPNs [11]. The Sud A group reported that first-degree relatives ofthe majority of patients with hematological malignancies exhibited increased familial relative risks(FRRs) for the same tumor type; the FRRs for MPN were >4.0, suggesting an inter relationship between FRRs and MPNs [22]. In our patients, *CHST15* mutation occurred with low frequencyin the same family member and was present in both germline and somatic cells, suggesting that *CHST15* germline mutation may be a founder lesion and is not accidental but instead is substantially interconnected. At the population level, a number of germline predisposition alleles have been identified for MPN, including rs2736100 in the *TERT* gene [8, 9], the *JAK2 46/1*haplotype [13, 14], and*RBBP6* germline mutation [12]. However, these data mainly came from Caucasian populations, and the universality of the mutations still remains to be evaluated, especially in Chinese populations. In the present study, we identified the *c.1367delG, p.Arg456fs*mutation in the *CHST15* gene as a predisposing genefor familial MPN. This conclusion was based on the following evidence: (i) the mutation is not found in the Exome Aggregation Consortium (ExAC) database (0/60, 706), indicating that it is very rare; (ii) there were no *CHST15* mutations in 110 patients with sporadic MPN and 160 healthy subjects from the same geographicalarea, excluding the possibility of polymorphism; and (iii) the previous generation (family 3, ET) and the subsequent generation (all three families) of the probands lacked the *CHST15* mutation (a total of 8 unaffected individuals), indicating that *CHST15* variant doesn’t follow a atavistic heredity models at multigenerational pedigree levels, and may be a de novo mutation.

Carbohydrate sulfotransferase 15 (*CHST15*) is a type II transmembrane glycoprotein that acts as a sulfotransferase and participates in chondroitin sulfate E (CS-E) biosynthesis [23]. CS-E plays a pivotal role in tumor progression [24] and tissue fibrosis formation [25, 26]. KatagiriBM T et al. assessed the effects of CS on hematopoiesis using a CS-reduced mouse model, in which a gene encoding the CS-synthesizing enzyme, CS N-acetylgalactosaminyltransferase-1 (CSGalNAcT1) was desrupted (T1KO), and BM levels of CS disaccharides in T1KO mice (930 pmol/mg acetone powder) were approximately two-thirds of those in WT mice (1400 pmol/mg acetone powder) while the expression level of CSGalNAcT1 in BM LSK cells (RT-qPCR) was very high, suggesting that immature hematopoietic cells are richer in CS disaccharides than mature ones. The hematopoietic stem and progenitor cells (HSPCs) from T1KO mice showed significantly impaired repopulation in WT recipient mice on serial transplantation., suggesting that CS is also important for HSC self-renewal, differentiation and homeostasis maintance in BM [27, 28]. Based on the impact of CSGalNAcT1 gene defect on hematopoietic, we speculate that CHST15 gene mutation might be involved in an insufficient CS-E synthesis, and participated in the abnormal hematopoietic in familial MPNs.

In addition, overexpression of *CHST15* may account for the prognosis of pancreatic cancer [29], invasive activity of breast cancer cells [30] and enhanced proliferation and sensitization to apoptosis of esophageal squamous cell carcinoma cells [31], suggesting the possibility that *CHST15* is a functional oncogene. Our results showed that familial MPN exhibited downregulated expression of both *CHST15* mRNA and *CHST15* protein. These characteristics suggest that the *CHST15* mutation endows MPN cells with increased proliferative activity and differentiation potential.

Coimmunoprecipitation experiment results indicated that *CHST15* may interact with *JAK2* both in HEL cells and in PBMNCs from familial MPN. The interaction was also confirmed by acquisition of immunofluorescence images, revealing for the first time that the physical interaction of *CHST15* with *JAK2* occurs in MPN cells, and suggested that a regulatory signaling axis, the *CHST15/JAK2/p-Stat3* axis, is involved in the pathogenesis of familial MPN.

To gain deeper insight into the molecular mechanisms and signaling pathways of *CHST15* mutation in familial MPN, gene expression profiling was performed. The results showed that *CHST15*-mutant cells had significant overexpression of the *FREM1,IFI27* and *C4B_2* genesand downregulated *AOC1* gene expression. These genes are mainly involved in the control of the inflammatory response, extracellular matrix formation and immune regulation,which contribute to a regulatory network supporting the generation of the inflammatory bone marrow microenvironment in PMF [32, 33]. An alternatively spliced *FREM1* transcript variant, named Toll-like/interleukin-1 receptor regulator (*TILRR*), is a coreceptor for members of the interleukin 1 receptor family. *TILRR* signaling contributes to the control of inflammation and amplifies *IL-1*-induced activation of *NF-κB* [34]. The *IFI27* gene is related to innate immune and interferon gamma signaling. In sporadic PMF patients, *IFI27* was overexpressed, reflecting the facilitation of PMF activity and enhancement of the tumor burden [35]. Although the *IFI27* gene was overexpressed in familial PMF, our coimmunoprecipitation did not confirm a direct interaction between *CHST15* and *IFI27*, suggesting both might exist in a signaling crosstalk. Complement signaling can influence both innate and adaptive immunity [36, 37], and both are involved in the development of inflammation-induced immune injury and stromareconstruction. We demonstrated that the *C4B_2* gene exhibited upregulated expression in familial PMF, implying that*C4B_2* may participate inthe development of local inflammatory lesions and the formation of extracellular matrix. *AOC1* may catalyze the degradation of putrescine and histamine. Polyaminesand their diamine precursor putrescine perform pivotal functions in cell growth. Kirschner et al. suggest that *Aoc1*gene expression contributes to the homeostasis of polyaminelevels in embryonic kidneys. Downregulation of *AOC1* is predicted to disturb the delicate balance of growth promotion and differentiation signals, thereby favoring a proliferative phenotype [38]. When the mRNA expression profiles of all six familial MPN pateints were integrated and hierarchical clustering analysis was performed, only two differentially expressed genes (*AOC1* and *NPDC1*) were identified. The *NPDC1* gene is able to suppress oncogenic transformation in neural and nonneural cells and overexpressed in intraductal papillary mucinous neoplasms of the pancreas [39] and soft tissue sarcoma [40]. Our patients exhibited upregulated NPDC1 expression, suggesting the existence of signaling communication between *CHST15* and *NPDC1*. When differentially expressed genes and proteins were integrated, we generated a signaling network related to the regulation of cell proliferation, differentiation, extracellular matrix formation and inflammatory responses associated with *CHST15* mutation in familial MPN, which was conducive to our understanding of the molecular mechanism of *CHST15* mutation-driven MPN.

In summary, analysis of three families with the same geographical origin identified a mutant locus in *CHST15* that is associated with the development of familial MPN. Further insights into the underlying mechanism of *CHST15* mutation-related MPN may lead to not only the identification of novel biomarkers but also the development of new therapeutic targets for MPN.

## Supplementary information

Supplementary data Revision 1

aj-checklist

Original Data File

## Data Availability

The authors confirm that the data supporting the findings of this study are available within the article and its supplementary materials.
