# Supplementary material for: CHST15 gene germline mutation is associated with the development of familial myeloproliferative neoplasms and higher transformation risk
Source: Cell Death Dis. 2022 Jul 7;13(7):586. doi: 10.1038/s41419-022-05035-w (PMC9263130; doi:10.1038/s41419-022-05035-w)
Supplement: Supplementary file 1 — Supplementary data Revision 1 [file 41419_2022_5035_MOESM1_ESM.docx]

**The Online Supplementary Methods**.

**(1)** **The colony formation experiment**

In briefly, HEL cells harbouring *CHST 15* mutant (1×10^5^) or wild-type *CHST15*(1×10^5^) were plated in 1 ml of RPMI-1640 methylcellulose culture medium in the presence or absence cytokines. The culture medium consist of 0.8% methylcellulose (Stem Cell Technologies, Vancouver, Canada), 1% deionised bovine serum albumin (BSA), 30% FCS and 2-β-mercaptoethanol (10−4 M). Culture dishes were incubated at 37°C for 14 d in a highly humidified atmosphere with 5% CO_22_. The colonies were enumerated using an inverted microscope. The experiment performed in triplicate.

**(2)** **RNA extraction, cDNA synthesis, and qRT-PCR**

Total RNA was extracted from fresh frozen PBMNCs using the TaKaRa MiniBEST Universal RNAExtraction Kit (Takara, 9767) according to the manufacturer’sinstructions. cDNA was synthesized using PrimeScript ™ RT reagent Kit with gDNA Eraser (Perfect Real Time) (Takara, RR047A). Real-time PCR was performed using TB Green™ Premix Ex Taq™ II (Tli RNaseH Plus)(Takara RR820A) and Light Cycler/Light Cycler 480 System(Roche Diagnostics) according to the manufacturer’s instructions. Expression levels of the target genes were normalized to that of β-actin. Primers sequences used were listed in Supplementary Materials (3).

**(3) Lentiviral vector constructs**

*CHST15*-mutant (*CHST15*^R456fs^) and wild-type *CHST15* (1638 bp) were amplified by RT-PCR from entry clone (*CHST15-WT*) or patient PBMNCs. Amplification products were purified by the Wizard SV Gel and PCR Clean-Up System (Promega) and verified by Sanger sequencing. Purified PCR products were cloned into the GV367 vector (AgeI/NheI), subcloned into a vector (pHelper2) expressing EGFP (pHelper1.0 and pHelper2.0 vectors) and cotransfected into HEL cells. Virus harvesting and identification were performed by Ji-Kai Genechem Co.,Shanghai, China. Cotransfected

HEL cells were grown in six-well plates in DMEM (Gibco-BRL,,, USA) supplemented with 10% FBS. When cells were 80% confluent, they were sequentially cultured for an additional 24 h, and further experiments were carried out.

**(4) Differentiation markers analysis of *CHST15* mutants**

HEL cells expressing wild-type or mutant *CHST15* were cultured in the presence or absence of cytokines, including stem cell factor (SCF: 50 ng/ml), interleukin-3(IL-3: 10 ng/ml), GM-CSF(50 ng/ml), G-CSF (50 ng/ml) and Epo (3U/ml), harvested and washed with phosphate-buffered saline (PBS) on ice, and then resuspended in FACS buffer. Antibodies against differentiation markers (CD14, CD15, CD61 and CD235a) were added and incubated for 1 h at 37°C in the dark. Flow cytometric data were collected and analyzed in a BD FACS Caliber using Cell Quest software.

**(5)Immunoblotting**

Protein expression levels in PBMNCs from patients with familial MPN or sporadic MPN and in HEL cells expressing the *CHST15* mutant was measured by immunoblotting. Protein signals were detected by chemiluminescence using SuperSignal reagent (Pierce, Rockford, IL).

**(6) Wright-Giemsa staining**

Cultured HEL cells were harvested, washed with PBS and fixed with 95% ice-cold methanol for 30 min at 4°C[19]. Cells were then seeded on slides and allowed to air dry. Next, cells were stained with Wright-Giemsa for 5 min and rinsed in deionized water. Finally, coverslips were fixed with Permount prior to microscopy (Carl Zeiss, Germany).

**(7)** **Immunohistochemical staining**

Bone marrow biopsy samples from familial PMF (n=4) and sporadic PMF (n=2) or healthy individuals (n=2) were formaldehyde fixed and paraffin embedded. Biopsy sections were dewaxed by heating at 65°C, and antigen retrieval was performed by heating in 10 mM citrate. Slides were incubated with a rabbit anti-human *CHST15* antibody overnight at 4°C and stained with HRP-labeled secondary antibodies. Staining was visualized and imaged under an Olympus microscope (40×).

**The Online Supplementary Materials**

(1) the *JAK2V617F* mutational burden analysis- allele-specific PCR

forward primer: 5'-AAGCTTTCTCACAAGCATTTGGTTT-3’

reverse primer: 5'-AGAAAGGCATTAGAAAGCCTG TAGTT-3'

Taqman probes specific for the wild type *JAK2* allele

VIC-5’-TCTCCACAGACACATAC- 3’MGB

or the mutant JAK2 allele

FAM-5’- TCCACAGAAACATAC-3’-MGB

(2)*CHST15* PCR(for sequencing): forward primer:5'- AAGCGGCTGGTGGTTTGTGT-3'

reverse primer:5'- CTGGGTGTCTTGCTAAATGG-3'

(3)*CHST15* reverse transcript PCR

forward primer: 5′-AACAACACCCTCAACAACGC-3';

reverse primer: 5′- CTGAAACGCAAACCCACAATG-3';

β-actin internal reference(RT-PCR)

forward primer:5'- TTCCAGCCTTCCTTCCTGGG-3

reverse primer:5'- TTGCGCTCAGGAGGAGCAAT-3

IFI27 （RT-PCR）

forward primer: 5'-CAGGATTGCTACAGTTGTGATTGG -3'

reverse primer:5'-CTATGGAGGACGAGGCGATTC-3'

AOC1 (RT-PCR)

forward primer: 5'-TCCACTACTATGATGCCGATGAC -3'

reverse primer:5'-ATTAAAGTGCCGCCGAAGGG-3'

C4B (RT-PCR)

forward primer: 5'-TTCACGAGGGCAAAGCAGAG -3'

reverse primer:5'-GCAATCCAGTAGGCAGACAGG-3'

FREM1(RT-PCR)

forward primer: 5'-AGGCTGGAGTTGTTCGCTATC -3'

reverse primer:5'-TGTGACGGATGCTGTGATGG-3'
