# Supplementary material for: CHST15 gene germline mutation is associated with the development of familial myeloproliferative neoplasms and higher transformation risk
Source: Cell Death Dis. 2022 Jul 7;13(7):586. doi: 10.1038/s41419-022-05035-w (PMC9263130; doi:10.1038/s41419-022-05035-w)

Original Data of Western Blotting

Figure S1. The original data of Figure 3C

STAT3


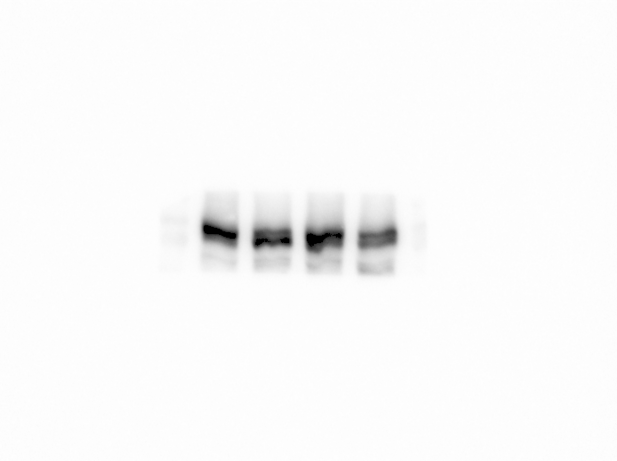


**79kDa**

**Extra**

**sample**

**86kDa**

**HEL**

**WT**

**MU**

**STAT3**

pSTAT3


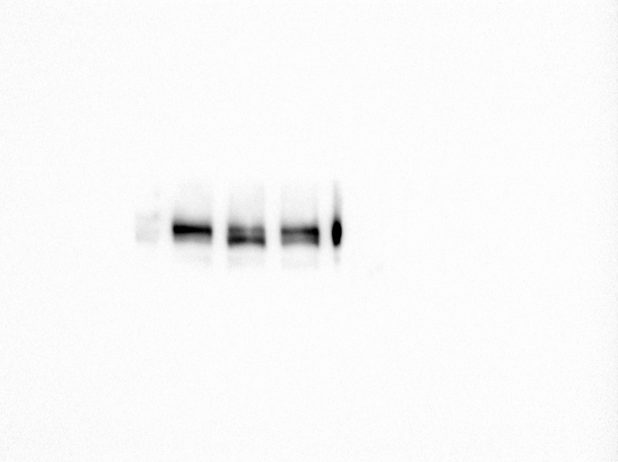


**79kDa**

**86kDa**

**Extra**

**sample**

**HEL**

**MU**

**WT**

**pSTAT3**

Figure S2. The original data of Figure 4A

**family**

**Control**

**Sporadic**


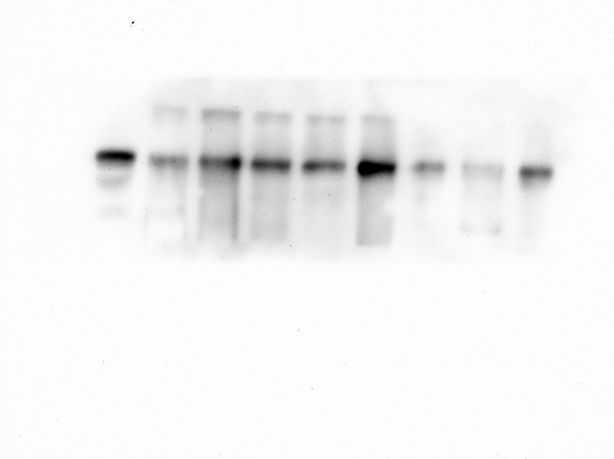


**65kDa**

**Extra**

**sample**

**1-1**

**2-1**

**1-2**

**2**

**1**

**1**

**3**

**2**

**CHST15**

Figure S3. The original data of Figure 4B

CHST 15

**Extra**

**sample**


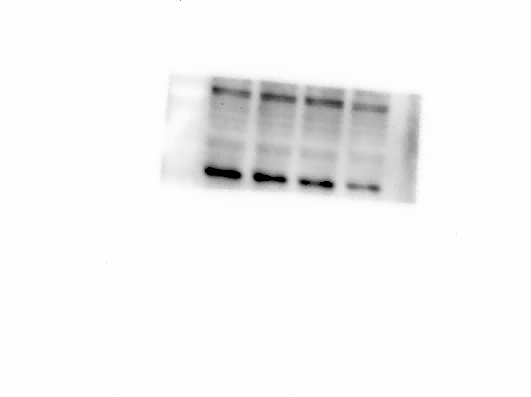


**65kDa**

**CHST15**

**HEL**

**Mut**

**WT**

β actin


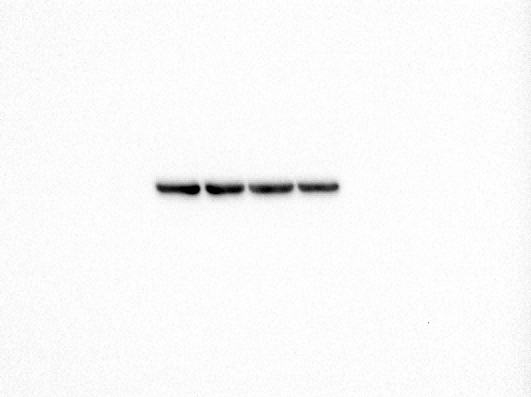


**45kDa**

**β actin**

**Extra**

**sample**

**HEL**

**Mut**

**WT**

Figure S4. The original data of Figure 6A

JAK2 (Input, Anti-JAK2，IgG)


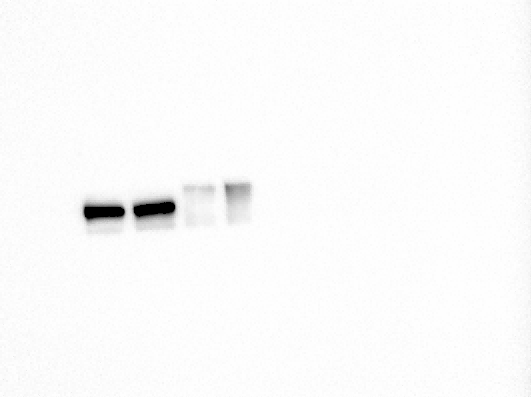


**125kDa**

**Input**

**Mut**

**IP**

**IgG**

**Anti-JAK2K**

**Mut**

**WT**

**WT**

**Mut**

**WT**

**JAK2**

CHST 15 (Input, Anti-JAK2，IgG)


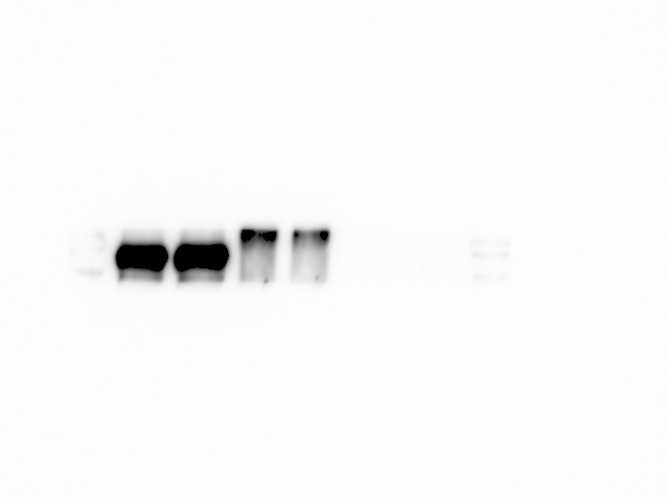


**65kDa**

**Mut**

**IP**

**Input**

**IgG**

**Anti-JAK2K**

**WT**

**Mut**

**WT**

**Mut**

**WT**

**CHST15**

3XFlag-CHST15 （Input, Anti-Flag, IgG）

**IP**

**3XFlag-CHST15**


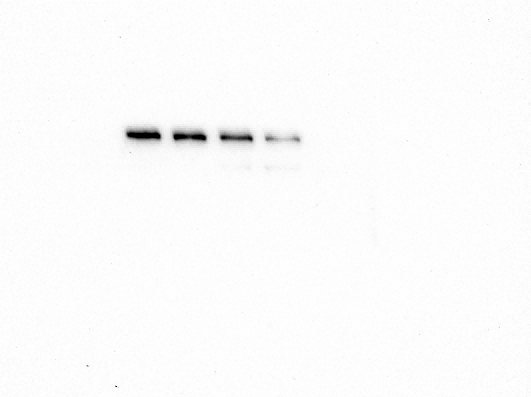


**65kDa**

**WT**

**Mut**

**WT**

**Mut**

**IgG**

**Anti-Flag**

**Input**

**WT**

**Mut**

JAK2 (Input, Anti-Flag, IgG)

**IP**


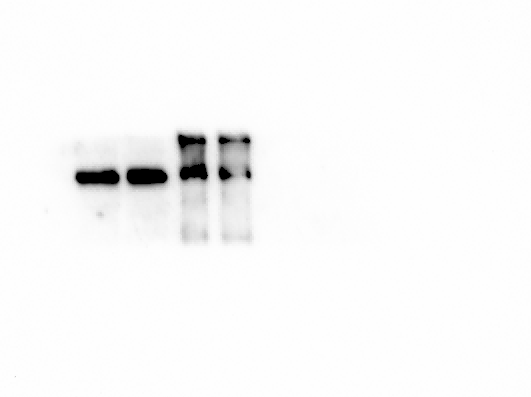


**125kDa**

**Input**

**Anti-Flag**

**IgG**

**Mut**

**WT**

**Mut**

**WT**

**WT**

**Mut**

**JAK2**

Figure S5 The original data of Figure 6B

JAK2 (Familial MPN)


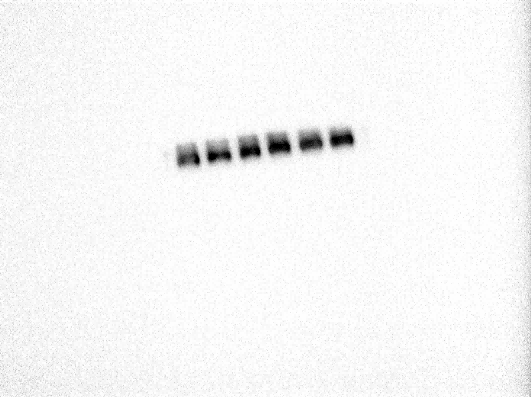


**125kDa**

**2**

**6**

**4**

**5**

**3**

**1**

**JAK2**

CHST 15 (Familial MPN)

Familial MPN


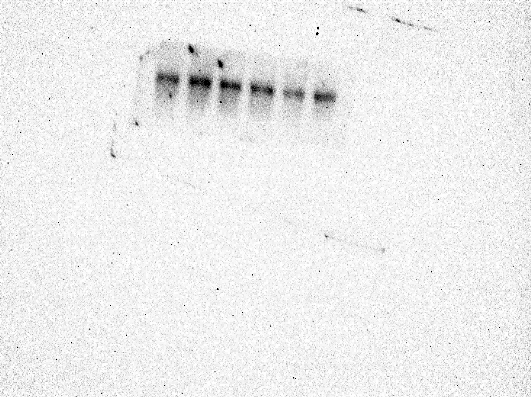


**65kDa**

**1**

**3**

**6**

**2**

**4**

**5**

**CHST15**

JAK2 (Sporadic MPN)


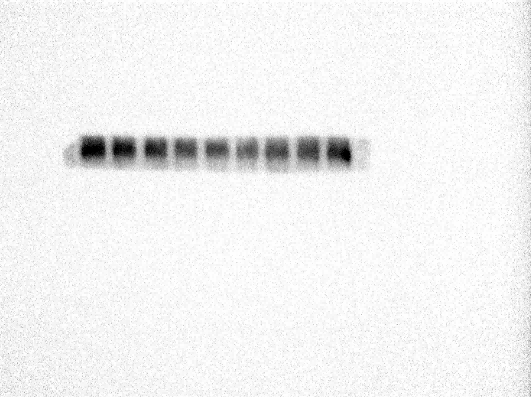


**125kDa**

Sporadic MPN

**9’**

**8’**

**7’**

**6’**

**5’**

**4’**

**3’**

**1’**

**2’**

**JAK2**

CHST15 (Sporadic MPN)


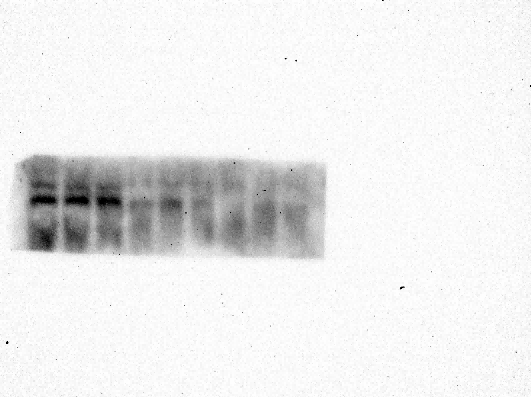


**CHST15**

**65kDa**

Figure S6 Real-time PCR of DEGs


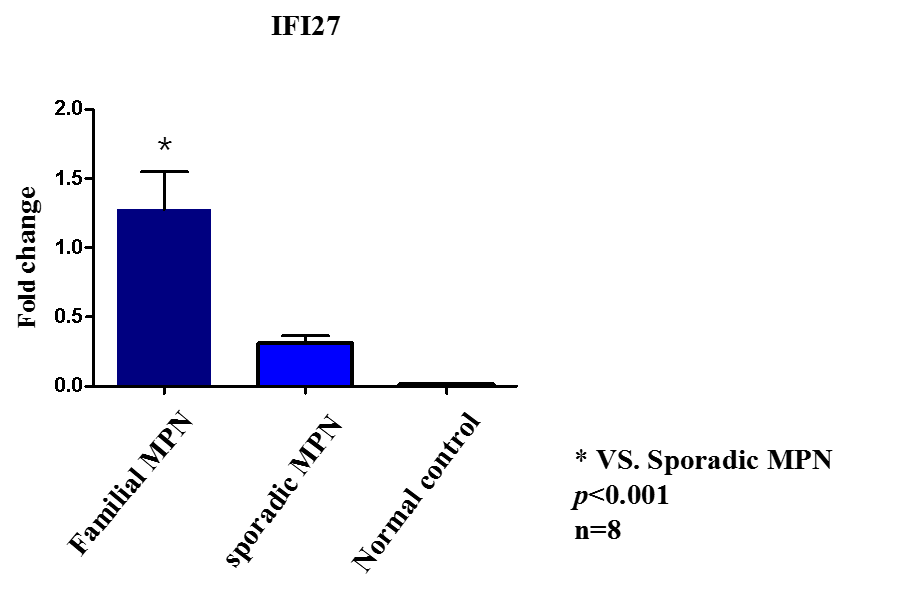

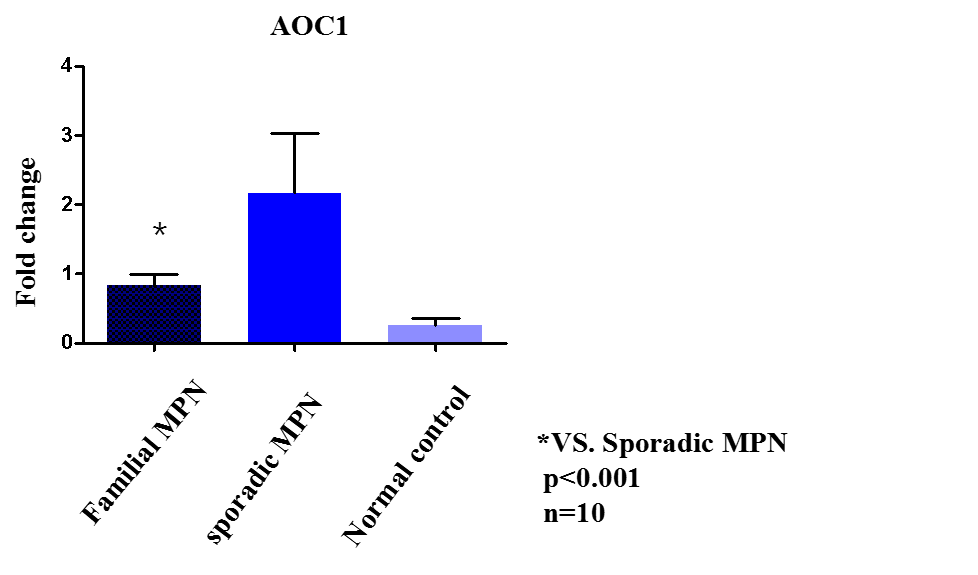


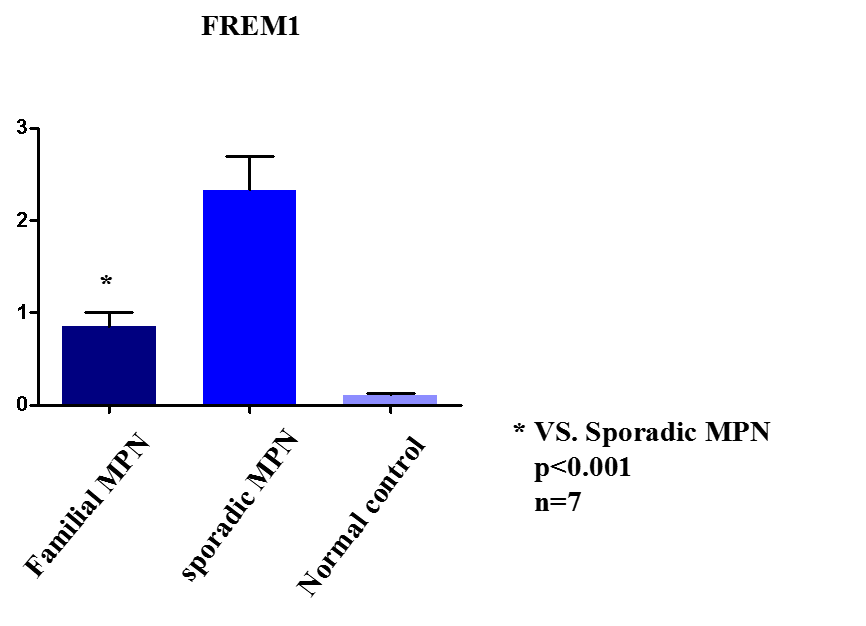

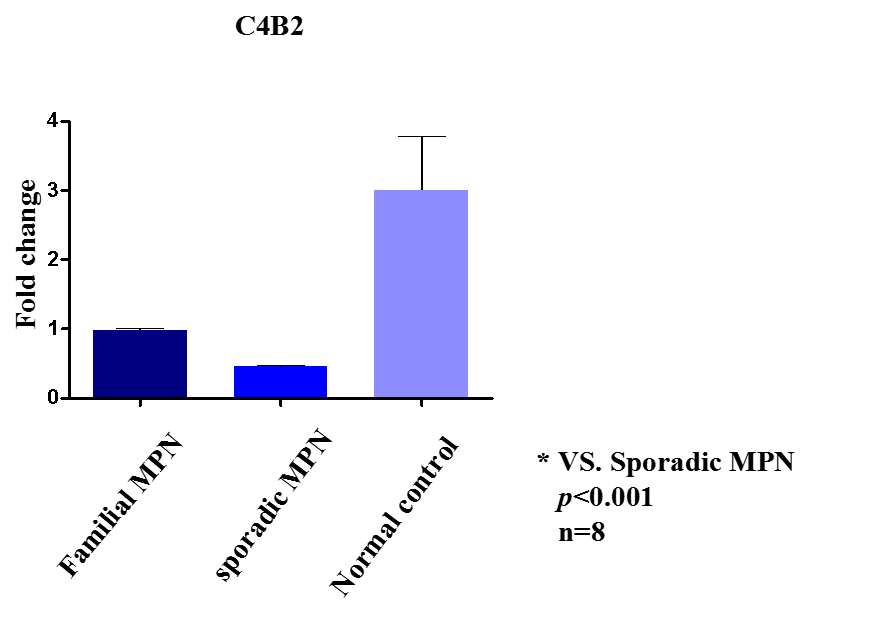

Supplement: Supplementary file 3 — Original Data File [file 41419_2022_5035_MOESM3_ESM.docx]
